# GA-TongueNet: tongue image segmentation network using innovative DiFP and MDi for stable generalization ability

**DOI:** 10.3389/fphys.2025.1617647

**Published:** 2025-06-24

**Authors:** Zhiyu Dong, Le Zhao, Yajun Fan, Haihua Ma, Changle Shao, Yiran Zhang, Peng Li

**Affiliations:** ^1^ College of Information Science and Engineering, Henan University of Technology, Zhengzhou, China; ^2^ Institute for Complexity Science, Henan University of Technology, Zhengzhou, China

**Keywords:** tongue segmentation, self-attention, transformer, dilated convolution, feature pyramid networks

## Abstract

Tongue is directly or indirectly connected to many internal organs in Traditional Chinese Medicine (TCM). In computer-aided diagnosis, tongue image segmentation is the first step in tongue diagnosis, and the precision of this segmentation is decisive in determining the accuracy of the tongue diagnosis results. Due to challenges such as insufficient available sample size and complex background, the generalization and robustness of current tongue segmentation algorithms are usually poor, which seriously hinders the practicality of tongue diagnosis. In this article, a GA-TongueNet, namely Tongue Segmentation Network for Stable Generalization Ability, based on self-attention architecture is proposed, which is a tongue segmentation network that can simultaneously have strong generalization ability and accuracy under small samples and diverse background conditions. Firstly, GA-TongueNet is built upon the transformer architecture, embedding the dilated feature pyramid (DiFP) module and the multi-dilated convolution (MDi) module proposed in this article. Secondly, the DiFP module is integrated to comprehend both the overall tongue image structure and intricate local details, while the MDi module is specifically designed to preserve a high feature resolution. Therefore, the network adeptly captures long-range dependencies, extracts high-level semantic content, and retains low-level detail information from tongue images. Moreover, it maintains decent precision and stable generalization capabilities, even when dealing with limited sample sizes. Experimental results show that the accuracy and generalization ability of GA-TongueNet in complex environments are significantly better than various existing semantic segmentation algorithms based on Convolutional Neural Networks (CNN) and Transformer architectures.

## 1 Introduction

As a widely accepted complementary and alternative medical approach, TCM has garnered increasing attention from the medical research community (Xu et al., 2020). Within the four diagnostic methods of TCM, tongue diagnosis constitutes a pivotal component of the observation procedure, serving as a cornerstone for clinical evaluation. By observing various characteristics of the tongue, such as its shape, color, and coating, practitioners can assess health conditions, the nature of diseases, and the functional state of internal organs (Wang et al., 2020). Rooted in TCM theory, the tongue serves as an external reflection of qi (vital energy) and blood circulation within the visceral systems, often referred to as the “visceral mirror”. This diagnostic approach provides an effective and non-invasive method for health assessment (Zhang et al., 2025). However, traditional tongue diagnosis relies heavily on empirical knowledge and subjective judgment, which may limit its reliability (Hu et al., 2019). With the rapid advancement of artificial intelligence in the medical field (Le et al., 2019), computer-aided tongue diagnosis has emerged as a promising avenue for addressing these limitations (Gao et al., 2022).

Computer-aided tongue diagnosis models typically rely on training and analysis of images captured by specialized tongue image acquisition devices (Cai et al., 2024). However, these images often include irrelevant facial or device-related information. Additionally, the inherent limitations of the acquisition devices lead to poor adaptability in diverse scenarios (Zhou et al., 2022). These challenges result in deviations in feature extraction and undermine the diagnostic reliability of such models (Qiu et al., 2023). Therefore, the development of a robust tongue image segmentation algorithm first enables precise extraction of critical pathological parameters including tongue substance and tongue coating by effectively separating the tongue body from extraneous background noise (Cao et al., 2023), and further serves as a crucial foundation for enhancing the accuracy and robustness of diagnostic models (Zhang et al., 2019).

The recent advancements in deep learning-related technologies have provided multiple research approaches for the tongue image segmentation task (Tng et al., 2022). Current mainstream methods are primarily based on CNN (Zhao et al., 2022), which leverage their powerful feature extraction capabilities to achieve notable success in semantic segmentation tasks and advance the field (Yu et al., 2021). However, CNN encounter inherent limitations when applied to complex natural environments (Monica et al., 2024). The restricted receptive field of convolution operations hinders their capacity to effectively capture contextual information (Liang et al., 2023). This limitation complicates the understanding of overall semantics and spatial relationships in tongue images, especially under diverse and challenging conditions (Feng et al., 2021). These limitations lead to poor generalization when dealing with tongue image data captured in varying acquisition environments, lighting conditions, and shooting angles. Additionally, CNN often struggle to accurately delineate the subtle edges of the tongue, resulting in segmentation precision that falls short of practical requirements (Li et al., 2021). The transformer architectures can effectively establish an integration mechanism for both local and global contextual information through self-attention mechanisms (Zhang et al., 2023), addressing the limitations of CNN and enhancing the model’s feature representation capabilities. However, these models impose substantial computational demands and require large-scale labeled datasets to prevent overfitting, as insufficient data often results in unstable convergence and compromised generalization performance.

To address these challenges, this article proposes GA-TongueNet, a novel model designed to enhance the generalization ability and stability of tongue image segmentation. Even when trained solely on datasets captured under standard acquisition scenarios, GA-TongueNet demonstrates high-precision boundary positioning capabilities and robust segmentation performance across diverse lighting conditions in natural environments. The key contributions of this article are summarized as follows:

• GA-TongueNet is proposed for tongue image semantic segmentation, effectively capturing long-range dependencies, high-level semantic information, and low-level detail information. The network achieves high precision and robust generalization even under complex backgrounds and small sample sizes.
• A novel DiFP module is proposed to better capture the overall structure and local details of tongue images, while the MDi module is designed to handle tongue images of varying sizes and maintain high feature resolution.
• Notably, GA-TongueNet achieves superior cross-domain generalization compared to Masked Autoencoders (MAE)-based methods without requiring pre-training, highlighting its inherent ability to learn discriminative features from limited data.

The remainder of this article is organized as follows: Section 2 reviews related work on tongue image segmentation. Section 3 details the proposed method, experimental materials and experimental results. Section 5 discusses the experimental results. Finally, Section 6 concludes the article.

## 2 Related work

### 2.1 Clustering-based methods

Clustering algorithms offer a promising approach for tongue image segmentation due to their ability to operate without heavy reliance on labeled data (Yuan et al., 2023). By automatically extracting and summarizing image features, they provide a flexible and versatile solution for segmenting biologically relevant structures in tongue images. For instance, to facilitate the automatic diagnosis of tongue images, Guo et al. (2016) proposed an automatic region segmentation algorithm that combines K-Means clustering with an adaptive activity contour network. Liu et al. (2018) improved the SLIC gamut distance formula, making the superpixels generated by SLIC more suitable for tongue image segmentation and reducing the segmentation time of the Grab Cut method. SGSCN (Ahn et al., 2021) iteratively learns the feature representation and cluster assignment for each pixel within a single image, while simultaneously ensuring that all pixels within a cluster remain spatially close to its center.

Despite their computational efficiency, clustering algorithms face inherent limitations in processing complex tongue images. The high variability in biological characteristics, such as texture, color, and morphology, can exceed the adaptability of these algorithms. Moreover, they are particularly vulnerable to noise interference, which may lead to substantial deviations in segmentation accuracy. These constraints diminish the reliability and robustness of clustering-based methods, making them less effective in addressing the nuanced demands of tongue image segmentation for biomedical applications.

### 2.2 CNN-based methods

CNN demonstrate strong feature extraction capabilities in processing tongue images, making them valuable for analyzing biological characteristics such as texture and color while preserving intricate details (Liu et al., 2022). For example, OET-NET (Huang et al., 2022) incorporates a residual soft connection module and a prominent image fusion module, coupled with a Focal Loss-based optimization strategy, to achieve effective tongue image segmentation in controlled environments. To address challenges associated with small sample sizes, QA-TSN (Jia et al., 2025) introduces a global rendering block to enhance global feature representation and employs modified partial convolution to accelerate real-time segmentation. Similarly, LAIU-Net (Marhamati et al., 2023) applies an optimized data augmentation strategy to segment biologically complex structures, such as sunken human tongues in photographic images. HPA-UNet (Yao et al., 2024) improves segmentation accuracy through enhanced data augmentation techniques and an updated U-Net architecture.

However, CNN-based methods encounter inherent limitations in addressing the complexities of tongue image segmentation, particularly when dealing with biological variability and challenging environmental conditions. These challenges include difficulty in segmenting small, biologically relevant structures, limited ability to capture global contextual features, and reduced generalization capabilities across diverse scenarios. Such limitations highlight the need for more robust and adaptable approaches to advance the segmentation of tongue images for biomedical applications.

### 2.3 Transformer-based methods

Transformers, with their self-attention mechanisms, offer a powerful framework for modeling relationships among different regions within an image. This characteristic is particularly beneficial for tongue image segmentation, where the accurate delineation of the tongue region is essential for analyzing biological features. Additionally, Transformers’ capacity for feature fusion enables the integration of multi-level information, enhancing segmentation accuracy. For instance, PriTongueNet (Huang et al., 2025) incorporates attention-guided skip connections and a self-distillation mechanism to address over-segmentation by supervising feature map differences during training. Similarly, Tongue-LiteSAM (Tan et al., 2025), a zero-shot model, achieves segmentation by integrating lightweight ViT-Tiny models based on the Segment Anything Model, providing a flexible approach for tongue image analysis. To minimize noise interference, Polyp-PVT (Wan et al., 2024) leverages the Swin-Transformer’s advanced feature extraction capabilities for analyzing sublingual veins, demonstrating its potential in capturing subtle biological details. In broader medical image segmentation, Slim UNETR (Pang et al., 2024) employs a decomposed self-attention mechanism to efficiently aggregate representations, achieving robust performance on resource-constrained devices.

While having advantages in capturing global features, transformers face limitations in modeling localized biological details. Their integration with other networks often encounters challenges in effectively balancing high-level semantic information with low-level structural details. Furthermore, Transformer-based architectures typically require large-scale training datasets to prevent overfitting. When applied to small-sample datasets, such as those often encountered in tongue image segmentation, the models may suffer from reduced generalization capability. This limitation is particularly pronounced in scenarios involving discontinuous tongue edges or complex backgrounds, where achieving fine-grained and biologically accurate segmentation remains a significant challenge.

## 3 Materials and methods

### 3.1 Tongue segmentation datasets

**FIGURE 1:**
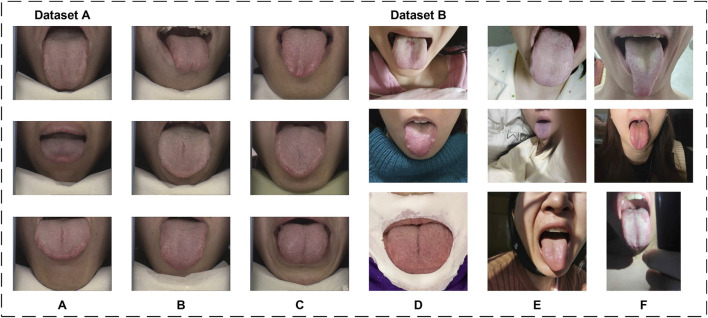
Sample images from the datasets. **(A–C)** are from Dataset A, **(D–F)** are from Dataset B.

The datasets utilized in this article consist of two subsets: Dataset A and Dataset B. Dataset A is used for both training and testing the model, while Dataset B is solely employed to evaluate the model’s generalization capability. Sample images from both datasets are shown in Figure 1.

#### 3.1.1 Dataset A

Dataset A originates from the publicly available BioHit (BioHit, 2014) dataset, comprising 300 tongue images with a resolution of 768×576 pixels. All images were collected using a standardized tongue imaging device, ensuring consistency in the positioning of the tongue across images. Corresponding ground truth annotations were meticulously prepared by experienced professionals, guaranteeing high-quality labeled data for model training.

#### 3.1.2 Dataset B

To further evaluate the generalization performance of the proposed model, we produced Dataset B by collecting 100 tongue images from public service tongue diagnosis posts on different online platforms. Tongue images were collected following stringent criteria to ensure diversity and realism in Dataset B. The collection guidelines excluded the use of filters, beauty enhancements, and identifiable features such as full facial images. Additionally, the dataset incorporates a variety of tongue-to-image size proportions, diverse lighting conditions, and multiple acquisition environments. These measures were designed to closely approximate real-world scenarios and enhance the dataset’s representativeness.

The labeling process utilized Labelme 5.5.0 to perform detailed segmentation annotations of the tongue body. According to TCM theory, different regions of the tongue correspond to various organs of the body. Therefore, the labeling approach adhered to a comprehensive standard, aiming to annotate all discernible parts of the tongue within the images. This included challenging areas such as the tongue root, which is often under-illuminated within the oral cavity. Efforts were made to ensure precise and thorough annotations, even in less visible regions.

### 3.2 The proposed method

In this section, the GA-TongueNet will be elaborated in detail. GA-TongueNet is inspired by the architecture of SegFormer (Xie et al., 2021) and employs a transformer-based encoder-decoder framework. Specifically, to ensure the trainability, convergence, and generalization ability of the model under small-sample conditions, we propose DiFP and MDi to enhance the model’s capability in perceiving and representing local detail features, global features, and contextual information.

#### 3.2.1 The architecture of GA-TongueNet

**FIGURE 2:**
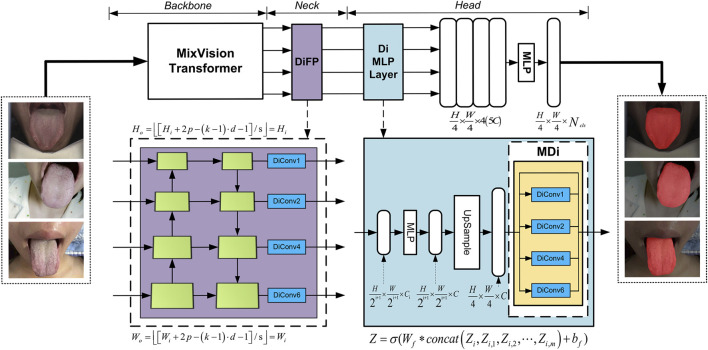
GA-TongueNet architecture.

The model proposed in this article comprises three key modules, as illustrated in Figure 2: the backbone for feature extraction, the neck for feature fusion, and the head for prediction. The backbone is based on the architecture of SegFormer and fully leverages the MixVision Transformer (MIT) module’s capability to extract multi-scale features.

For the neck module, we introduce targeted structural enhancements, resulting in the development of the DiFP module. This module efficiently fuses multi-scale feature information and, more importantly, deeply captures contextual information without compromising resolution. Such capabilities significantly contribute to precise segmentation of tongue details, thereby improving both the granularity and generalization ability of the segmentation results.

In the head module, we innovatively propose the MDi and incorporate it into the MLP layer. This design allows the model to develop a deeper understanding of the global structure within images, enabling it to effectively handle tongue images of varying sizes. Consequently, this enhances the model’s adaptability and generalization performance, ensuring accurate and robust segmentation across diverse scenarios.

#### 3.2.2 The construction of the DiFP module

The Feature Pyramid Networks (FPN) (Lin et al., 2017) have demonstrated outstanding capabilities in processing multi-scale features. However, its performance encounters certain limitations when applied to precise pixel-level prediction tasks. To construct a high-performance multi-level feature map structure capable of efficiently handling multi-scale objects while maintaining high feature map resolution, we meticulously improved the original FPN module to develop the DiFP, which serves as the neck component of our model.

**FIGURE 3:**
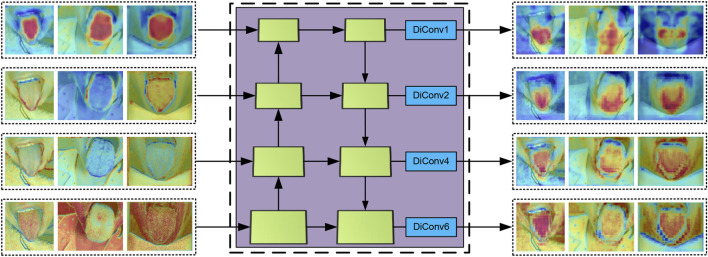
The input and output feature maps of DiFP.

Building upon the original FPN, the DiFP integrates the key technology of dilated convolution. Specifically, 3×3 dilated convolutions with varying dilation rates d replace the original standard 3×3 convolution operations, thereby enhancing the smoothness of the feature maps. Figure 3 illustrates the input and output feature maps of the module. The core innovation of dilated convolution lies in its ability to expand the receptive field effectively without significantly increasing the number of parameters, achieved by strategically inserting gaps between the convolution kernel elements. This is further explained in Formula 1 for the receptive field.

$$Receptive\;Field=\left(k-1\right)\times d+1$$ (1)

where k is the size of the convolution kernel, and d is the different dilation rates.

Formula 2 ensures that the spatial resolution of the output feature map remains consistent with the input after the application of dilated convolution, which is critical for preserving resolution in pixel-level prediction tasks. In the proposed DiFP, the dilation rates d are set to 1, 2, 4, and 6 to effectively capture features at varying receptive fields. To counterbalance the increased spatial requirements introduced by dilated convolutions, the padding p is configured to match the dilation rate d . Furthermore, with the kernel size k fixed at 3 and stride s set to 1, the structural integrity and resolution consistency of the feature maps are maintained throughout the process.

$$\left\{\begin{array}{cc} H_{o} & =\left\lfloor \frac{H_{i}+2p-\left(k-1\right)\cdot d-1}{s}\right\rfloor =H_{i}\\ W_{o} & =\left\lfloor \frac{W_{i}+2p-\left(k-1\right)\cdot d-1}{s}\right\rfloor =W_{i} \end{array}\right.$$ (2)

where, $H_{o}$ and $W_{o}$ denote the height and width of the output feature map, respectively, while $H_{i}$ and $W_{i}$ represent the height and width of the input feature map. The parameter p corresponds to the padding applied, s refers to the stride, and ⌊⌋ indicates the downward rounding operation.

The DiFP method effectively captures the target’s multi-scale features, enabling the processing of objects at varying scales. Additionally, it preserves the resolution of the feature map, thereby enhancing prediction accuracy in fine-grained tasks while ensuring robust generalization performance across diverse data distributions.

#### 3.2.3 The construction of the MDi module

To enable the model to accurately capture contextual information at multiple scales and extract finer details, we developed the MDi module and integrated it into the Di MLP layer. By combining dilated convolutions with both large and small dilation rates while retaining the original feature map, this design effectively enhances the model’s ability to capture both local and global features. With a simple structure that relies on basic connections and 1×1 convolutions for feature fusion, the MDi module improves computational efficiency, enhances sensitivity to complex scenes, and boosts performance in boundary detection tasks.

**FIGURE 4:**
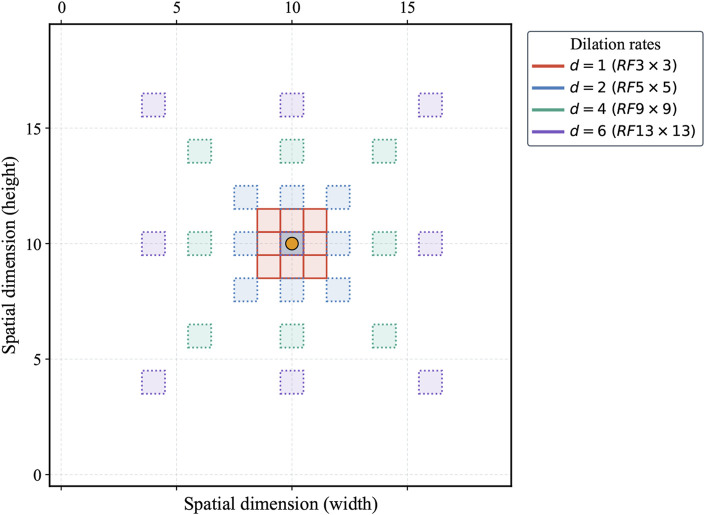
Multi-scale dilated convolution in MDi module. RF represents receptive fields.

As illustrated in Figure 2, the MDi module employs a multi-scale dilated convolution mechanism to generate multi-scale feature maps. Specifically, each input feature map is processed through m dilated convolution layers, resulting in m+1 feature maps, where the additional map corresponds to the original input feature. The colored grid in Figure 4 represents the multi-scale receptive field, demonstrating the module’s ability to balance local and global feature extraction effectively. Dilated convolutions with smaller dilation rates excel at capturing fine-grained boundary details, ensuring that subtle features are preserved. In contrast, larger dilation rates allow the module to extract broader contextual information, contributing to a more comprehensive understanding of the overall scene.

After multi-scale feature fusion, the MDi module produces a combined feature map, mathematically expressed in Equation 3. This fused feature map integrates local details with global structural features of the tongue image. The fusion process, visually represented as a colored grid overlay, highlights how the expanded receptive field enables accurate and comprehensive segmentation. By achieving a balanced representation of fine and coarse features, the MDi module significantly improves the model’s segmentation performance.

$$Z=\sigma \left(W_{f}*concat\left(Z_{i},Z_{i,1},Z_{i,2},\ldots ,Z_{i,m}\right)+b_{f}\right)$$ (3)

where $Z_{i}$ is the result of the *i*th input feature map after passing through a 1×1 convolution and upsampling. $Z_{i,j}$ is the result of the *i*th input feature map after passing through the *j*th dilated convolution. $W_{f}$ is the weight matrix for the 1×1 convolution used for feature fusion, $b_{f}$ is the bias term for the 1×1 convolution, and σ is the activation function.

### 3.3 Implementation details

The models utilized in this study were developed and implemented within the framework of mmsegmentation 1.2.2, with the exception of RTC_TongueNet (Tang et al., 2024) and TongueSAM (Cao et al., 2023), which were evaluated using their officially recommended configuration environments to ensure optimal performance. All other models were implemented using Python 3.8.2 and PyTorch 2.1.0, with computations powered by CUDA 11.8 on an ASUS TUF Gaming FX507VV platform (CPU: Intel Core i7-13700H; GPU: NVIDIA GeForce RTX 4060, 8 GB). The Dataset A, comprising 300 tongue images, was randomly divided into a training set (270 images) and a test set (30 images). During training, a variety of data augmentation techniques were applied to enhance the model’s robustness and adaptability to different input conditions. These techniques included scaling (resizing with a factor range of 0.5–2.0), cropping (retaining a random 75% area of the image), flipping with a probability of 0.5, and adjusting brightness (ranging from −32 to +32), contrast (ranging from 0.5 to 1.5), and saturation (ranging from 0.5 to 1.5). These augmentation strategies ensured that the model was exposed to a wide range of variations during training, enhancing its robustness and generalization performance across diverse datasets and input scenarios.

## 4 Results

### 4.1 Evaluation metrics

To assess the performance of the proposed model, four commonly used tongue segmentation evaluation metrics are employed: Dice, IoU, Precision, and Recall. Dice, as shown in Equation 4, denotes the metric of overlap between two sets, reflecting the accuracy of tongue target extraction. IoU, as shown in Equation 5, defined as the ratio of intersection to concatenation, intuitively reflects the accuracy of the segmentation results. Precision, as shown in Equation 6, refers to the ratio of the number of correctly predicted positive samples among all the predicted positive samples, which reflects the reliability of the prediction results. Recall, as shown in Equation 7, refers to the ratio of the number of correctly predicted positive samples to the total number of true positive samples, which evaluates the completeness of tongue segmentation. Higher values of the above four indicators mean better segmentation performance of the model. The formulas are as follows:

$$Dice=\frac{2\times \left|X\cap Y\right|}{\left|X\right|+\left|Y\right|}$$ (4)

$$IoU=\frac{\left|X\cap Y\right|}{\left|X\cup Y\right|}$$ (5)

where X and Y denote the sets of predicted and ground truth pixels, respectively.

$$Precision=\frac{TP}{TP+FP}$$ (6)

$$Recall=\frac{TP}{TP+FN}$$ (7)

where TP , FP and FN denote true positives, false positives and false negatives, respectively.

### 4.2 Methods comparison

**FIGURE 5:**
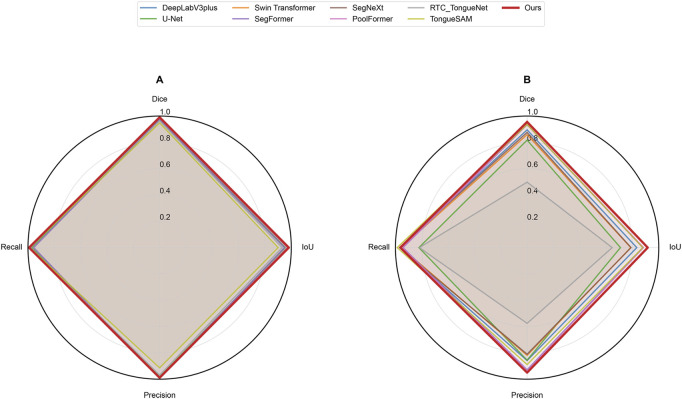
Radar chart of evaluation metrics for different networks on datasets. **(A)** represents Dataset A and **(B)** represents Dataset B.

**FIGURE 6:**
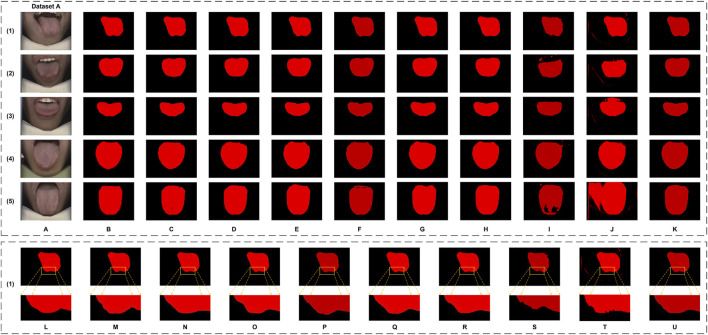
The tongue segmentation results of different networks on Dataset A. **(A)** represents the original image, **(B)** represents the ground truth, and **(C–K)** represent U-Net, DeepLabV3plus, Swin Transformer, SegFormer, SegNeXt, PoolFormer, RTC_TongueNet, TongueSAM, and Ours, respectively. **(L–U)** are the local magnification images, using (1) as an example from Dataset A, and show the ground truth, U-Net, DeepLabV3plus, Swin Transformer, SegFormer, SegNeXt, PoolFormer, RTC_TongueNet, TongueSAM, and Ours, respectively.

**FIGURE 7:**
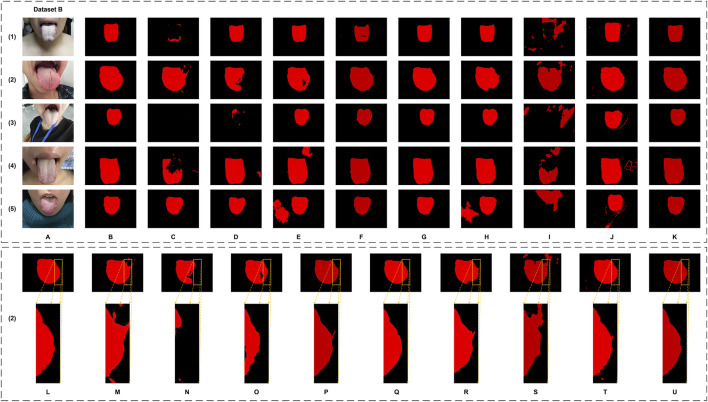
The tongue segmentation results of different networks on Dataset B. **(A)** represents the original image, **(B)** represents the ground truth, and **(C–K)** represent U-Net, DeepLabV3plus, Swin Transformer, SegFormer, SegNeXt, PoolFormer, RTC_TongueNet, TongueSAM, and Ours, respectively. **(L–U)** are the local magnification images, using (2) as an example from Dataset B, and show the ground truth, U-Net, DeepLabV3plus, Swin Transformer, SegFormer, SegNeXt, PoolFormer, RTC_TongueNet, TongueSAM, and Ours, respectively.

In this study, the performance of the proposed GA-TongueNet was comprehensively compared with eight other models. These include six well-established semantic segmentation models: DeepLabV3plus (Chen et al., 2018), U-Net (Ronneberger et al., 2015), Swin Transformer (Liu et al., 2021), SegFormer (Xie et al., 2021), SegNeXt (Guo et al., 2022), and PoolFormer (Yu et al., 2022), as well as two recently developed models specifically designed for tongue image segmentation: RTC_TongueNet (Tang et al., 2024) and TongueSAM (Cao et al., 2023). The sizes and inference speeds of these models are summarized in Table 1. To evaluate their performance in a standard acquisition environment, all models were trained and tested on Dataset A. Additionally, to assess their generalization capability, further tests were conducted on Dataset B, which comprises more challenging and diverse scenarios. The evaluation metrics include Dice, IoU, Precision, and Recall. Quantitative results for both Dataset A and Dataset B are presented in Table 2 and are visually summarized in the radar chart shown in Figure 5. Furthermore, representative segmentation outcomes from the two datasets are illustrated in Figures 6, 7, providing qualitative comparisons that highlight the strengths and weaknesses of the various models.

**TABLE 1:** Model size and inference speed of different networks.

Network	Parameters (M)	FPS
DeepLabV3plus (Chen et al., 2018)	41.22	13.35
U-Net (Ronneberger et al., 2015)	28.99	3.84
Swin Transformer (Liu et al., 2021)	41.64	11.50
SegFormer (Xie et al., 2021)	0.89	60.33
SegNeXt (Guo et al., 2022)	4.26	43.42
PoolFormer (Yu et al., 2022)	15.634	58.79
RTC_TongueNet (Tang et al., 2024)	55.42	5.99
TongueSAM (Cao et al., 2023)	102.67	3.36
Ours	14.01	11.77

**TABLE 2:** Evaluation metrics data for different networks.

Dataset	Network	Dice	IoU	Precision	Recall
Dataset A	DeepLabV3plus (Chen et al., 2018)	0.9851	0.9706	0.9879	0.9823
	U-Net (Ronneberger et al., 2015)	0.9765	0.9541	0.9827	0.9704
	Swin Transformer (Liu et al., 2021)	0.9863	0.9729	0.9885	0.9841
	SegFormer (Xie et al., 2021)	0.9734	0.9481	**0.9909**	0.9565
	SegNeXt (Guo et al., 2022)	0.9890	0.9781	0.9873	0.9906
	PoolFormer (Yu et al., 2022)	0.9845	0.9694	0.9865	0.9824
	RTC_TongueNet (Tang et al., 2024)	0.9640	0.9310	0.9691	0.9850
	TongueSAM (Cao et al., 2023)	0.9476	0.9005	0.9141	0.9883
	Ours	**0.9906**	**0.9814**	0.9894	**0.9918**
Dataset B	DeepLabV3plus (Chen et al., 2018)	0.8953	0.8334	0.8585	0.9681
	U-Net (Ronneberger et al., 2015)	0.8171	0.7092	0.8560	0.8219
	Swin Transformer (Liu et al., 2021)	0.8587	0.7874	0.8190	0.9567
	SegFormer (Xie et al., 2021)	0.9339	0.8784	0.9289	0.9423
	SegNeXt (Guo et al., 2022)	0.8757	0.7895	0.8103	0.9697
	PoolFormer (Yu et al., 2022)	0.9358	0.8848	0.9349	0.9440
	RTC_TongueNet (Tang et al., 2024)	0.4975	0.6463	0.5759	0.8184
	TongueSAM (Cao et al., 2023)	0.9356	0.8790	0.8893	**0.9870**
	Ours	**0.9553**	**0.9163**	**0.9501**	0.9632

The bold values represent the optimal values.

#### 4.2.1 Performance analysis

The model size and inference speed of GA-TongueNet are moderate, as detailed in Table 1. Its design strikes a balance, positioning it as neither particularly lightweight nor excessively resource-intensive. Among the nine evaluated models, GA-TongueNet demonstrated competitive performance, surpassing the two models specifically designed for tongue image segmentation. Notably, it achieved the optimal performance in tongue segmentation, underscoring its superior effectiveness in this specialized task.

On Dataset A, GA-TongueNet achieved a Dice of 0.9906 and an IoU of 0.9814, outperforming widely used CNN-based models such as DeepLabV3plus (Dice: 0.9851, IoU: 0.9706), U-Net (Dice: 0.9765, IoU: 0.9541), and SegNeXt (Dice: 0.9890, IoU: 0.9781). It also demonstrated advantages over Transformer-based models, including Swin Transformer (Dice: 0.9863, IoU: 0.9729), SegFormer (Dice: 0.9734, IoU: 0.9481), and PoolFormer (Dice: 0.9845, IoU: 0.9694). While GA-TongueNet’s Precision (0.9894) was slightly lower than SegFormer’s (0.9909), its Dice (0.9906), and IoU (0.9814) Recall (0.9918) were the highest among all evaluated models. Furthermore, compared to RTC_TongueNet (Dice: 0.9640, IoU: 0.9310) and TongueSAM (Dice: 0.9476, IoU: 0.9005), both of which are specifically designed for tongue image segmentation, GA-TongueNet demonstrated superior performance across all evaluation metrics. As illustrated in Figure 5A, while the performances of the compared models are generally comparable, GA-TongueNet exhibits a more outward trajectory on the radar chart, reflecting its relatively superior overall performance in this task.

On the more challenging and diverse Dataset B, GA-TongueNet demonstrates clear advantages. As shown in Figure 5B, the overall performance of GA-TongueNet is notably superior among the nine evaluated models. Particularly for metrics such as Dice and IoU, GA-TongueNet exhibits a more pronounced outward trajectory, reflecting its relatively superior performance. The model achieved a Dice of 0.9553, IoU of 0.9163, Precision of 0.9501, and Recall of 0.9632, outperforming all competing models. For instance, compared to DeepLabV3plus (Dice: 0.8953, IoU: 0.8334), GA-TongueNet shows improvements of 6.70% in Dice and 9.88% in IoU. Similarly, its IoU exceeds that of U-Net (IoU: 0.7092) and Swin Transformer (IoU: 0.7874) by 20.71% and 12.89%, respectively, highlighting its capability to generalize effectively to complex real-world data. Even when compared to the strong Transformer-based competitor SegFormer, GA-TongueNet achieves better results, with Dice, IoU, Precision, and Recall being 0.0214, 0.0379, 0.0212, and 0.0209 higher, respectively. These results underscore the robustness and adaptability of GA-TongueNet across varying data distributions. Although SegNeXt performed commendably on Dataset A, it encountered significant challenges in generalizing to the complex conditions of Dataset B, achieving an IoU of only 0.7895, substantially lower than GA-TongueNet. Similarly, while PoolFormer achieved a relatively high IoU of 0.8848, it remained 3.16% lower than that of GA-TongueNet. The two models specifically designed for tongue image segmentation, RTC_TongueNet and TongueSAM, also lagged behind GA-TongueNet in comprehensive performance. Notably, RTC_TongueNet exhibited relatively poor generalization ability. These findings highlight the limitations of traditional CNN-based architectures and some Transformer-based designs in addressing the complexities of diverse and challenging scenarios, while reinforcing the stable generalization and adaptability of GA-TongueNet.

Qualitatively, as illustrated in Figure 6, GA-TongueNet demonstrates high-precision boundary delineation on Dataset A. The detailed segmentation performance of each model is further highlighted in Figures 6L–U, where local details are examined. While most models exhibit segmentation results that align well with the ground truth, the performance of RTC_TongueNet and TongueSAM shows room for improvement, particularly given the constraints of the current small-scale dataset. In contrast, Figure 7 reveals GA-TongueNet’s robustness in handling challenging conditions such as varying lighting and complex backgrounds, scenarios that prove difficult for other models. From Figures 7L–U, it becomes evident that under uneven illumination, U-Net and DeepLabV3plus struggle with issues of false detection and incomplete region segmentation. Swin Transformer performs admirably in standard acquisition environments but fails to generalize effectively to Dataset B. SegFormer, despite being competitive, encounters challenges such as boundary recognition errors. Similarly, while SegNeXt and PoolFormer exhibit strong performance, they remain slightly inferior to GA-TongueNet in terms of accuracy and consistency. For the tongue-specific models, RTC_TongueNet displays limited generalization ability, making precise segmentation in complex environments challenging. TongueSAM performs relatively better, achieving successful segmentation for most tongue bodies; however, it also exhibits a higher rate of false positives. These comprehensive results underscore GA-TongueNet’s ability to achieve accurate and reliable tongue segmentation while maintaining adaptability across diverse and complex data environments. Its superior generalization and robustness further highlight its potential as a dependable tool for tongue image segmentation in real-world applications.

#### 4.2.2 Comprehensive analysis for methods comparison

Under the constraints of small-sample datasets, the experimental results demonstrate that GA-TongueNet achieves remarkable segmentation performance, across both standard and challenging scenarios. Its comprehensive performance surpasses that of current comparison models, highlighting its strong generalization capability and robustness. GA-TongueNet’s ability to handle complex backgrounds and diverse conditions effectively makes it particularly well-suited for applications involving limited training data. This adaptability underscores its potential for reliable deployment in both standard acquisition environments and more complex, real-world scenarios.

### 4.3 Ablation study

**FIGURE 8:**
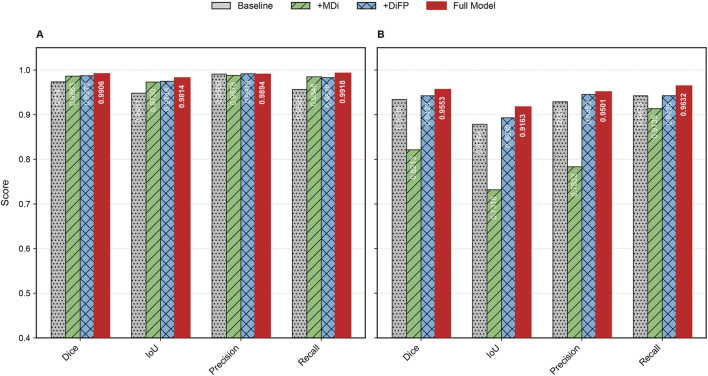
Bar chart of evaluation metrics for ablation study on datasets. **(A)** represents Dataset A and **(B)** represents Dataset B.

To thoroughly investigate the contributions of the proposed components to the overall performance of the model, we conducted an ablation study. Specifically, we compared the SegFormer baseline model (Baseline), the model enhanced with the MDi module (+MDi), the model integrated with the DiFP module (+DiFP), and the model incorporating both MDi and DiFP modules (Full Model). The experiments were conducted on both Dataset A and Dataset B, using Dice, IoU, Precision, and Recall as evaluation metrics to rigorously assess the effectiveness of the improvement strategies. The results are detailed in Table 3, and visually represented in Figure 8.

**TABLE 3:** Evaluation metrics data for ablation study.

Dataset	Network	Dice	IoU	Precision	Recall
Dataset A	Baseline	0.9734	0.9481	0.9909	0.9565
	+MDi	0.9863	0.9730	0.9878	0.9848
	+DiFP	0.9872	0.9748	**0.9916**	0.9829
	Full Model	**0.9906**	**0.9814**	0.9894	**0.9918**
Dataset B	Baseline	0.9339	0.8784	0.9289	0.9423
	+MDi	0.8213	0.7318	0.7832	0.9135
	+DiFP	0.9423	0.8930	0.9453	0.9425
	Full Model	**0.9553**	**0.9163**	**0.9501**	**0.9632**

The bold values represent the optimal values.

#### 4.3.1 Performance analysis

**FIGURE 9:**
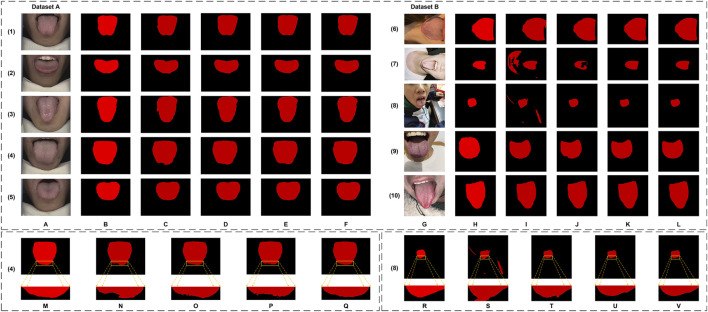
The tongue segmentation results of the ablation study on datasets. **(A–F)** are from Dataset A. **(A)** represents the original image, **(B)** represents the ground truth, and **(C–F)** represent Baseline, +MDi, +DiFP, and Full Model, respectively. **(G–L)** are from Dataset B. **(G)** represents the original image, **(H)** represents the ground truth, and **(I–L)** represent Baseline, +MDi, +DiFP, and Full Model, respectively. **(M–Q)** are the local magnification images from Dataset A, using (4) as an example, and show the ground truth, Baseline, +MDi, +DiFP, and Full Model, respectively. **(R–V)** are the local magnification images from Dataset B, using (8) as an example, and show the ground truth, Baseline, +MDi, +DiFP, and Full Model, respectively.

The introduction of the MDi and DiFP modules has significantly influenced model performance on both Dataset A and Dataset B, demonstrating complementary effects, as shown in Table 3; Figure 9. On Dataset A, the addition of the MDi module improves the Dice and IoU to 0.9863 and 0.9730, respectively, compared to the Baseline model (Dice: 0.9734, IoU: 0.9481). The DiFP module further enhances the performance, achieving a Dice of 0.9872 and an IoU of 0.9748. When both modules are integrated in the Full Model, the performance reaches its peak, with a Dice of 0.9906 and an IoU of 0.9814. These results highlight the ability of the DiFP module to capture detailed structural features and the role of the MDi module in maintaining feature resolution across varying tongue sizes. Notably, as illustrated in Figures 9M–Q, the segmentation results of the Full Model align more closely with the ground truth, reducing errors observed in the Baseline and single-module configurations. In particular, Figures 9N–P show false positives.

On Dataset B, the performance trend reflects different behaviors under more complex conditions. The Baseline model achieves satisfactory results, with a Dice of 0.9339 and an IoU of 0.8784. However, the inclusion of the MDi module unexpectedly leads to a decrease in performance, with Dice and IoU dropping to 0.8213 and 0.7318, respectively. This decline suggests that the MDi module introduces instability in handling diverse and challenging scenarios. In contrast, the +DiFP model performs better, achieving a Dice of 0.9423 and an IoU of 0.8930, which surpasses the Baseline performance. When MDi and DiFP are integrated, the Full Model demonstrates the best generalization capability, achieving a Dice of 0.9553, an IoU of 0.9163, a Precision of 0.9501, and a Recall of 0.9632. These results underscore the complementary effects of the two modules in improving segmentation performance under challenging conditions. Figures 9R–V further corroborates these findings. In Dataset B’s challenging scenarios, Figures 9S, T show false positives. The Full Model achieves more accurate segmentation of the tongue region, minimizing both false positives and missed areas.

#### 4.3.2 Comprehensive analysis for ablation study

The ablation study reveals the complementary contributions of the MDi and DiFP modules to the overall model performance. Dataset A, serving as the training set and characterized by relatively standardized data, shows consistent improvements when either module is added individually. The Full Model, which integrates both MDi and DiFP, achieves the highest scores across all evaluation metrics, indicating effective synergy between these components.

In contrast, Dataset B, used exclusively as a testing set and representing more complex and diverse scenarios, exhibits different behavior. The MDi module alone results in a drop in performance compared to the Baseline, with the IoU decreasing from 0.8784 to 0.7318. This suggests that MDi, when applied independently, may introduce instability or reduced robustness in challenging environments. The DiFP module performs more reliably on Dataset B and improves several metrics over the Baseline, though it does not fully surpass it on all measures. Importantly, the combined Full Model leverages the complementary strengths of MDi and DiFP to achieve superior performance, demonstrating better generalization and robustness on Dataset B despite its complexity.

These results indicate that while the MDi module may have limitations when deployed independently on unseen complex data, its integration with the DiFP module provides a more balanced and stable architecture. The synergy between these modules enhances the model’s ability to generalize from training on Dataset A to challenging test scenarios in Dataset B, thereby improving segmentation accuracy and robustness in practical applications.

### 4.4 Generalization verification

To verify the generalization ability of GA-TongueNet, we first used the 95% confidence interval (CI) and 5-fold cross-validation. Secondly, we took the MAE with excellent generalization ability as the backbone of our model to further verify the generalization ability.

#### 4.4.1 Preliminary verification

Comparison experiments and ablation studies indicate that GA-TongueNet, when trained on the standard environment of Dataset A, achieves superior performance in tongue segmentation when applied to the more complex and natural scenarios of Dataset B. Preliminary validation of the generalization ability by up to 5-fold cross-validation trained yielded stable and good metrics: Dice (98.95 ± 0.06%), IoU (97.92 ± 0.12%), Precision (98.88 ± 0.19%), and Recall (99.08 ± 0.15%). These results reflect the robustness and consistency of the model, and to a certain extent, rule out the possibility of overfitting. In addition, in terms of CI, we first calculated the individual metrics (e.g., IoU, Dice, Precision, Recall) for each of the 30 images in Dataset A and 100 images in Dataset B. The mean and variance of these metrics were then determined, providing the basis for deriving the final CI. From the experimental results, Dataset A performs well as Dice 98.68% (95% CI [97.18%, 97.62%]), IoU 97.40% (95% CI [98.57%, 98.79%]), Precision 99.35% (95% CI [99.11%, 99.58%]) and Recall 98.04% (95% CI [97.78%, 98.29%]). In comparison, while the performance on Dataset B shows slight degradation, the metrics remain robust: Dice 95.53% (95% CI [94.85%, 96.21%]), IoU 91.63% (95% CI [90.46%, 92.81%]), Precision 95.01% (95% CI [94.03%, 95.99%]) and Recall 96.32% (95% CI [95.48%, 97.16%]). As shown in Figure 1, Dataset B is extremely different from Dataset A in terms of lighting, background, and shooting angle, which somewhat validates the model’s potential for real-world application in unseen environments.

#### 4.4.2 Further verification

The MAE framework has been shown to enhance model generalization through mechanisms such as unsupervised learning, high-ratio masking, and latent representation learning (He et al., 2022). While the comparative and ablation experiments presented earlier effectively demonstrate the generalization ability of the original GA-TongueNet structure (the proposed model in this study), we conducted further verification by integrating MAE as the backbone. Specifically, MAE was pre-trained on a dataset of 4,213 tongue images, 70% of which were augmented versions of Dataset A (using techniques such as rotation and color transformation), while the remaining images resembled those in Dataset B.

**FIGURE 10:**
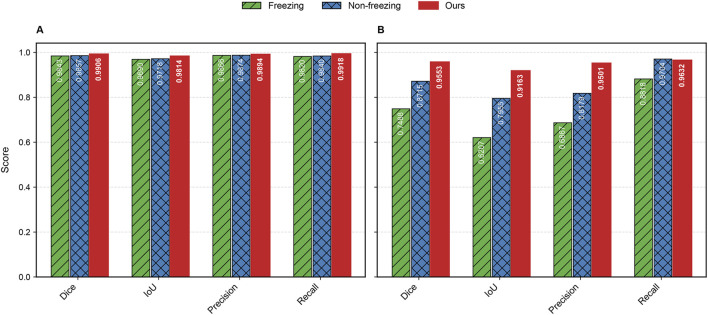
Bar chart of evaluation metrics for generalization verification on datasets. **(A)** represents Dataset A and **(B)** represents Dataset B.

**FIGURE 11:**
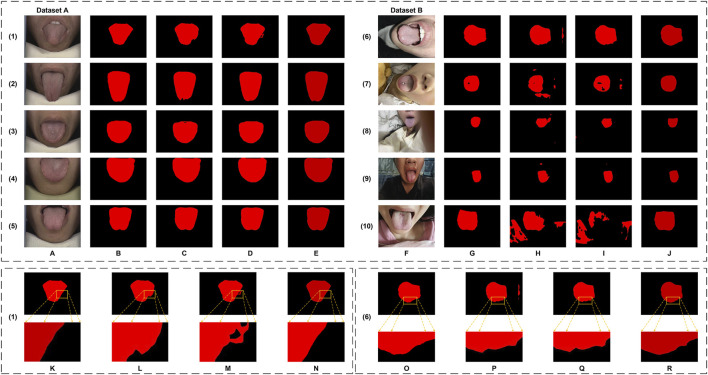
The tongue segmentation results of generalization verification on datasets. **(A–E)** are from Dataset A. **(A)** represents the original image, **(B)** represents the ground truth, and **(C–E)** represent Freezing, Non-freezing, and Ours, respectively. **(F–J)** are from Dataset B. **(F)** represents the original image, **(G)** represents the ground truth, and **(H–J)** represent Freezing, Non-freezing, and Ours, respectively. **(K–N)** are the local magnification images from Dataset A, using (1) as an example, and show the ground truth, Freezing, Non-freezing, and Ours, respectively. **(O–R)** are the local magnification images from Dataset B, using (6) as an example, and show the ground truth, Freezing, Non-freezing, and Ours, respectively.

Following this pre-training, we applied two strategies—Freezing and Non-Freezing—for training GA-TongueNet with MAE as the backbone. These experiments were designed to strengthen the evidence supporting the generalization capability of the original GA-TongueNet structure. The experimental results for these models, including the original GA-TongueNet, and the MAE-based variants trained using Freezing and Non-Freezing strategies, are presented in Table 4; Figures 10, 11.

**TABLE 4:** Evaluation metrics data for generalization verification.

Dataset	Network	Dice	IoU	Precision	Recall
Dataset A	Freezing	0.9843	0.9690	0.9866	0.9820
	Non-Freezing	0.9857	0.9718	0.9874	0.9840
	Ours	**0.9906**	**0.9814**	**0.9894**	**0.9918**
Dataset B	Freezing	0.7488	0.6207	0.6867	0.8818
	Non-Freezing	0.8715	0.7955	0.8179	**0.9704**
	Ours	**0.9553**	**0.9163**	**0.9501**	0.9632

The bold values represent the optimal values.

The evaluation metrics for Dataset A and Dataset B are summarized in Table 4. For Dataset A, the proposed original GA-TongueNet shows consistent improvements over the MAE-based GA-TongueNet under both Freezing and Non-Freezing strategies. Across all metrics, including Dice, IoU, Precision, and Recall, the original GA-TongueNet achieves the highest scores, with Dice of 0.9906, IoU of 0.9814, Precision of 0.9894, and Recall of 0.9918. These results suggest that the architecture is well-suited for handling the relatively controlled conditions in Dataset A.

For Dataset B, the Non-Freezing strategy outperforms the Freezing strategy, achieving a Dice score of 0.8715, IoU of 0.7955, Precision of 0.8179, and Recall of 0.9704. This indicates that allowing parameter adjustments during training can help the model adapt better to the diverse and complex scenarios in Dataset B. Nonetheless, the performance of the Non-Freezing strategy remains below that of the original GA-TongueNet. The proposed original GA-TongueNet achieves Dice, IoU, Precision, and Recall scores of 0.9553, 0.9163, 0.9501, and 0.9632, respectively, on Dataset B. Compared to the Non-Freezing strategy, these values represent noticeable improvements in Dice (9.6%), IoU (15.1%), and Precision (13.2%), while Recall is marginally lower by 0.7%. These findings suggest that the original GA-TongueNet is capable of addressing complex scenarios in Dataset B effectively, while the slight trade-off in Recall indicates potential areas for further refinement. Overall, the results indicate that the proposed model achieves balanced performance across datasets with varying complexity, demonstrating a degree of robustness and adaptability.

Figure 11 provides a visual comparison of the segmentation results across the two datasets. In Dataset A, while both the Freezing and Non-Freezing strategies demonstrate reasonable performance, as shown in Figures 11L,M, challenges remain in handling edge details, with occasional false positives observed. For Dataset B, the performance of both strategies diminishes in the presence of complex scenarios, such as foreign objects like tongue studs. As illustrated in Figures 11P,Q, these approaches face difficulties in effectively addressing edge detail segmentation under such conditions. In contrast, original GA-TongueNet demonstrates improved robustness, providing more accurate segmentation of the tongue body while minimizing significant false positives or omissions. These observations highlight GA-TongueNet’s potential for enhanced performance in varied and challenging environments, though opportunities for further improvement remain.

#### 4.4.3 Comprehensive analysis for generalization verification

The experimental results indicate that in preliminary evaluations, the proposed GA-TongueNet demonstrated favorable performance, providing an initial validation of its generalization ability. Further testing revealed that, compared to its MAE-backbone variant, the original GA-TongueNet exhibited superior generalization and robustness. On Dataset A, characterized by relatively standard conditions, the original GA-TongueNet leveraged its architectural design to achieve high segmentation performance. On Dataset B, which features more complex backgrounds and diverse variations, the model demonstrated stronger adaptability by effectively mitigating interference and maintaining consistent segmentation quality. The performance gap between the original GA-TongueNet and the MAE-backbone variant was particularly pronounced in challenging scenarios, underscoring the potential advantages of the proposed architecture. These findings support the conclusion that the original GA-TongueNet possesses commendable generalization capabilities. By effectively addressing diverse and complex conditions, including unseen environments, the original GA-TongueNet demonstrates promise as a reliable tongue image segmentation model for practical applications.

## 5 Discussion

In TCM, tongue features are considered key indicators of an individual’s physiological functions and pathological changes. However, in computer-aided diagnosis, the accuracy of tongue diagnosis can be significantly affected by confounding factors such as teeth, facial regions, and other background elements. Precise tongue image segmentation is therefore critical for enhancing diagnostic accuracy.

From a clinical perspective, accurate tongue image segmentation is fundamental to improving the performance of computer-assisted tongue diagnosis systems, particularly in mobile applications. As illustrated in Dataset B of Figure 1, most tongue images captured in real-world scenarios often include complex backgrounds. Without proper segmentation, the surrounding environment of the tongue body introduces substantial noise, which can compromise the accuracy of the analysis. Segmentation isolates the tongue body, allowing diagnostic models to focus exclusively on relevant features without being influenced by extraneous factors. This targeted approach significantly enhances the precision and reliability of tongue diagnosis.

Tongue segmentation is crucial for extracting disease-related features, such as the color of the tongue body and the distribution and thickness of the tongue coating. According to TCM theory, these features are strongly correlated with the functional states of internal organs. For instance, variations in the thickness and color of the tongue coating may reflect digestive system abnormalities or indicate internal dampness or heat, providing valuable insights for syndrome differentiation and treatment planning. Additionally, region-specific analysis of the tongue—such as the tip, center, and base—enables a nuanced understanding of organ-specific functions. For example, a red or yellow coating on the tongue tip may suggest hyperactivity of heart fire, while a greasy coating on the tongue base could indicate renal insufficiency. These correlations underscore the diagnostic value of precise tongue segmentation. The robustness of segmentation algorithms is equally critical for their application in diverse clinical environments. Variations in lighting conditions, background interference, and differences in tongue posture can introduce significant variability in tongue images. A well-designed segmentation model capable of addressing these challenges ensures consistent and accurate image analysis, irrespective of the imaging environment. This consistency is vital for generating reliable diagnostic data across populations and regions. By overcoming these challenges, tongue segmentation significantly enhances the clinical utility of computer-assisted tongue diagnosis, enabling applications such as early disease detection, comprehensive health status evaluation, and effective monitoring of treatment outcomes.

With the rapid advancement of deep learning techniques, CNN, renowned for their robust feature extraction capabilities, have been widely applied in tongue segmentation. Notable models include OET-NET (Huang et al., 2022), QA-TSN (Jia et al., 2025), LAIU-Net (Marhamati et al., 2023), and HPA-UNet (Yao et al., 2024). In addition, models leveraging the self-attention mechanism of Transformers, such as PriTongueNet (Huang et al., 2025) and Tongue-LiteSAM (Tan et al., 2025), have demonstrated promising segmentation performance. However, these models often require extensive training datasets and exhibit limited generalization ability.

In comparison, our proposed GA-TongueNet demonstrates high segmentation accuracy despite relying on a relatively modest training dataset. It utilizes the publicly available Dataset A (BioHit, 2014), consisting of 300 tongue images, and an additional 100 images collected from diverse and complex contexts for generalization testing. Comparative experiments against four representative models—spanning both CNN and Transformer architectures—demonstrate that GA-TongueNet consistently outperforms these models. It effectively addresses the challenges associated with limited and heterogeneous datasets, showcasing its potential to alleviate existing limitations in tongue image segmentation tasks.

To further explore the generalization potential of GA-TongueNet, we integrated a pre-trained MAE (He et al., 2022) as its backbone. MAE, known for its proficiency in unsupervised learning, high-rate masking, and latent representation learning, was trained on a large-scale tongue image dataset. However, experimental results reveal that this modified architecture underperforms relative to the original GA-TongueNet. This unexpected outcome underscores the inherent adaptability and robustness of the original GA-TongueNet design, which maintains high segmentation accuracy even for tongue images outside the training set.

Although the results are encouraging, there are still some deficiencies in this study. Judging from the experimental results, GA-TongueNet may make false positives when facing unfamiliar situations, such as foreign objects on the tongue (such as punctured tongue nails, tongue perforations), and mistakenly think it is the tongue body. For areas with poor lighting, such as the back of the tongue, false positives may occur. Furthermore, in the fine segmentation of the tongue edge, the result is not always optimal. This is more obvious at the boundary between the tongue and adjacent structures such as teeth or lips, where the division may appear blurred and less precise. As for the model itself, although the current model has achieved the best segmentation effect in experiments, the computational cost is not the lowest. It requires higher computing resources and a longer time. How to reduce computing costs and computing resources has become one of the directions we are actively improving, hoping to be deployed on more devices. Furthermore, the MDi module and the DiFP module need to work in synergy to achieve the optimal performance.

## 6 Conclusion

In this article, we have presented GA-TongueNet, a model designed for tongue image segmentation, addressing the challenges of semantic segmentation in complex environments. By incorporating the DiFP and MDi modules, the model demonstrates the ability to achieve multi-scale feature fusion and effectively capture contextual information. Despite being trained only on tongue images obtained in standard acquisition environments, GA-TongueNet shows promising performance in segmenting images captured under challenging lighting conditions.The experimental results suggest that GA-TongueNet performs well in terms of segmentation accuracy and generalization across diverse and complex environments. While there is still potential for improvement, these findings indicate that GA-TongueNet could serve as a useful approach for tongue image segmentation in real-world applications.

## Data Availability

Publicly available datasets were analyzed in this study. This data can be found here: https://github.com/BioHit/TongeImageDataset.
